# Processing genome scale tabular data with wormtable

**DOI:** 10.1186/1471-2105-14-356

**Published:** 2013-12-05

**Authors:** Jerome Kelleher, Rob W Ness, Daniel L Halligan

**Affiliations:** 1 University of Edinburgh, King’s Buildings, West Mains Road, Edinburgh, EH9 3JT, UK

## Abstract

**Background:**

Modern biological science generates a vast amount of data, the analysis of which presents a major challenge to researchers. Data are commonly represented in tables stored as plain text files and require line-by-line parsing for analysis, which is time consuming and error prone. Furthermore, there is no simple means of indexing these files so that rows containing particular values can be quickly found.

**Results:**

We introduce a new data format and software library called wormtable, which provides efficient access to tabular data in Python. Wormtable stores data in a compact binary format, provides random access to rows, and enables sophisticated indexing on columns within these tables. Files written in existing formats can be easily converted to wormtable format, and we provide conversion utilities for the VCF and GTF formats.

**Conclusions:**

Wormtable’s simple API allows users to process large tables orders of magnitude more quickly than is possible when parsing text. Furthermore, the indexing facilities provide efficient access to subsets of the data along with providing useful methods of summarising columns. Since third-party libraries or custom code are no longer needed to parse complex plain text formats, analysis code can also be substantially simpler as well as being uniform across different data formats. These benefits of reduced code complexity and greatly increased performance allow users much greater freedom to explore their data.

## Background

Despite the ever increasing volumes of data being processed in bioinformatics, the methods used are almost entirely based on plain text files. Data is usually encoded in lines of text, with each row consisting of a series of tab-delimited values. These files are easy to view and interpret and can be processed on any platform with the minimum of library dependencies. Using compression, text files can be quite compact, and specialised indexing methods are available to retrieve specific rows, for example rows which intersect with a given genomic interval [1].

It is not sufficient, however, to simply store and retrieve data. To use data it must be *processed*. This is the major flaw in using text files as a data format; before we can perform calculations, we must first parse the encoded information into native machine values. This is a computationally expensive process, and compression (if it is used) adds substantial overhead. As a result, simple calculations over a large dataset may take many hours to complete.

Another problem with tables stored as text files is that it is difficult to index the information in particular columns. This means that many operations on a table require a complete scan through the file. The only viable means of working with a subset of the data, therefore, is to create another file consisting of the subset of interest. This is inflexible and error prone, and multiplies already significant storage requirements.

The obvious solution to these problems is to load tabular data into a relational database. Databases store values in binary form so that parsing is not required, and support efficient retrieval and indexing. However, there are many problems with this approach. Relational databases are complex systems, each supporting different features and SQL dialects. It is not a straightforward task to design a schema for a particular dataset, particularly not if portability across different databases is required. Similarly, accessing data requires a knowledge of SQL. In the most common case, a database server must be maintained and so storage and user permissions must be carefully managed. All of these aspects require significant expertise which is why so many researchers and programs continue to use textual data formats.

A relational database server is far more than we require in the majority of cases. Data files are usually written once and not subsequently expected to change. Thus, storing this information in a relational database with its sophisticated concurrency control is entirely unnecessary. Centralised storage of datasets creates an unnecessary administration burden, as does an extra layer of user management. Therefore, although relational databases provide powerful data management technologies, they are complex to use and maintain and are rarely used for static datasets.

## Implementation

Wormtable (write-once read-many table) is a new data format and software library designed specifically to deal with the challenges of data processing in bioinformatics. It provides a portable, compact, read-only store for tabular data of essentially unlimited size. Tables can be either written directly using the library, or converted from existing formats via command line tools. Wormtable provides a simple, user friendly Python API to access rows in the table, facilitating efficient and convenient access to data. The core data manipulation and storage facilities are written in C for efficiency.

Data is stored in rows using a compact binary format. Each row consists of a defined number of columns, and each column holds elements of a given type and size. Each column is allocated a fixed number of bytes within the ‘fixed region’ of a row. For columns with fixed length, the encoded elements are stored entirely within the fixed region; for columns of variable length, the encoded elements are stored in the ‘variable region’ of the row, and the location and number of elements stored in the fixed region. The type, size and number of elements of each column are specified in the table schema, which is defined in XML as part of the table’s metadata.

Wormtable currently supports signed and unsigned integers, floating point and character data. Integer sizes range from one to eight bytes, and real numbers are stored as IEEE half, single, and double precision [2] floating point values. Fixed and variable length character strings are also supported. Using this flexible type system, columns can be assigned the most appropriate type and size for the range of values to be represented.

Rows are stored sequentially in a data file, and the offset and length of each row is stored in a Berkeley DB [3] database. Thus, any given row can be found by first looking up the database for its offset and length, and by then reading the required set of bytes from the data file. Berkeley DB is a free and open-source embedded database toolkit that provides a scalable key-value store. It is a mature and stable platform, and is currently the most widely deployed database toolkit in the world [4].

Besides random access to rows, wormtable also provides indexes over arbitrary combinations of columns. An index is a Berkeley DB database in which the keys are the elements from the columns in question concatenated together, and the values are pointers to the original rows. Indexes make many operations much more efficient, because we can go directly to the rows we are interested in without performing a full table scan. They also provide a very efficient means of calculating a histogram for a given set of columns. Moreover, wormtable supports binned indexes, in which a range of values is mapped to a single index key. This is very useful for floating point columns, where it may not be necessary to distinguish between very similar values.

Each table corresponds to a directory in the file system which is used to store the data files, indexes and metadata. Files within a table’s home directory are not intended to be manipulated directly by users, and the wtadmin program is provided to perform administration tasks such as adding and removing indexes. This approach is very flexible, since no centralised storage is required and tables can be moved around and between systems at will. Tables are portable across operating systems and hardware architectures.

## Results

### API

The principle goal of wormtable is to provide efficient access to data using an easy to learn interface. In Python, the Table class provides the main interface, and implements the standard Python sequence protocols. Efficient iteration over rows is provided by the cursor method, which takes as an argument the list of columns to read. Only the values for the columns of interest are then retrieved, leading to considerable time savings. The cursor method can take two additional arguments, start and stop, which specify the rows of interest. This allows us to efficiently seek to an arbitrary location in the table and to read a given number of rows sequentially from this point.

The Index class also has a cursor method with the same signature, but in this case, rows are returned in the order defined by the index. The start and stop arguments are now defined in terms of index keys: all rows in which the index key is greater than or equal to start and less than stop are returned. Partial keys may also be provided for multi-column indexes. The Index class also provides an iterator over all keys, as well as a means of counting the number of rows with a given key.

The API is straightforward, but it is flexible, powerful and extensible. This simplicity ensures that programmers of all experience levels can take full advantage of the powerful data processing facilities that wormtable provides. In the following subsections we illustrate the performance advantages of wormtable via some examples. These are not intended to be definitive benchmarks but are simple examples to demonstrate the type of improvements that can be expected by using wormtable over existing methods.

### Scan performance

The Variant Call Format (VCF) encodes information about variant sites in a genome as tab-delimited rows in a text file [5]. VCF is one of the most commonly used formats to store genomic data from next generation sequencing. To illustrate the advantages of wormtable when performing calculations over a whole table, we converted a large publicly available VCF file (produced as part of the Drosophila genetic reference panel [6]) to wormtable format. This VCF [7] consists of 15GB of uncompressed text and contains data from the whole genome over 6,146,611 rows. Using the included vcf2wt program, the VCF was converted to wormtable format on a workstation with an Intel Xeon processor, 12GB of RAM and a single hard disk. The conversion required approximately 69 minutes, and the size of the resulting wormtable was 10GB (using the smallest type required to represent the data in each column).

Values are stored in wormtable in a portable binary format, so that no parsing is required when reading in rows. To illustrate this advantage, we wrote a script to count the number of transitions and transversions in the dataset using wormtable and PyVCF [8], a Python VCF parser. In this example we proceed row-by-row, examining the REF and ALT columns and counting the transitions and transversions we encounter. Using PyVCF this required approximately 126 minutes, whereas the wormtable version required 57 seconds. To compare against methods that are known to be extremely efficient, we repeated the same example using the Unix tools cut, grep and awk, which required 80 seconds. Both the Unix pipeline and wormtable were limited by the sequential read bandwidth of the hard drive, and would therefore be much faster using modern solid state storage. It should be noted, however, that although Unix pipelines are efficient, it is a difficult and error prone method of processing data when more complex calculations are required.

Full table scans are often unnecessary in wormtable. For many tasks, creating an appropriate index allows us to seek directly to the rows of interest. To illustrate this, we repeated the example of counting transitions and transversions using an index on the REF and ALT columns. The index required 3m40s to build and consumed 66MB of storage space. Counting the number of transitions and transversions using this index required less than a second.

### Seek performance

One of the most serious problems with tabular data stored in text files is that it is not possible to access a particular row efficiently without some auxiliary index. Unless we have some information on where a particular row is located in a file, there is little that can be done except to read the file line-by-line until the required row is found. Tabix [1] solves this problem by compressing a tab-delimited text file into blocks, and then storing an index mapping genome position to the location of the compressed block, and the position of the row within the block. Tabix is specifically designed for range queries, allowing us to efficiently retrieve all of the rows within a given genomic range.

To compare the seek performance of wormtable with Tabix, we compressed the VCF file mentioned in the previous subsection using bgzip (which required 11m8s) and indexed it with tabix (1m12s). The size of the resulting compressed file was 2.9GB and the size of the index file was 106KB. To duplicate the functionality of Tabix on VCF data, we simply need to create an index on the CHROM and POS columns using wtadmin add. This required approximately 2 minutes and the resulting index consumed 115MB of space.

We compared the seek performance of Tabix and wormtable by generating a genomic location randomly and retrieving all rows within 1Kb of this location. This was repeated 10^4^ times, and we measured both the elapsed and processor time. The Tabix Python module was used for the comparison, ensuring that no overheads associated with process forking were incurred. The same set of random locations were used for wormtable and Tabix, ensuring a fair comparison.

When using a cold cache (i.e., no pages of the files in question are present in the operating system’s cache) the elapsed time for Tabix was 103 seconds with a processor time of 65 seconds. For wormtable, the elapsed time was 181 seconds with a processor time of 5 seconds. Thus, the time required to perform this test is dominated by waiting for I/O in both cases. Since the wormtable file is considerably larger than the compressed file used by Tabix, more and larger hard drive seeks were required to bring the required pages into memory. Once the pages were in memory, however, Tabix needed to do much more work to decompress then and make them usable, as shown by the difference in processor times.

This difference is well illustrated by immediately repeating the same experiment, so that all the relevant pages are in cache. In this case, the elapsed time was 56 seconds for Tabix and around 2 seconds for wormtable. Thus, the use of compression is a trade-off: it reduces file size, which reduces the number of random seeks required, but decompression is expensive and must be repeated each time a block is accessed. The advantages of a smaller file in terms of reducing the number of seeks incurred would also be largely negated by using solid state storage, where random seeks do not incur such a heavy penalty.

The test used here is also highly synthetic, and unlikely to be indicative of most real-world applications. The majority of workloads have strong locality of reference [9], and such large and extreme jumps across genomic regions are unlikely to occur. In this case, wormtable is much faster than Tabix, since there is no CPU overhead of decompression. Furthermore, since Tabix returns rows of text, the problem of parsing rows must still be solved. This is expensive (as illustrated in the previous subsection), and creates extra code complexity. Wormtable, by providing a simple API to access both rows and columns, gives a unified interface for accessing data that is both straightforward to use and highly efficient.

## Discussion

The problems of enabling efficient random access to rows and avoiding the large overhead of parsing text are well understood, and efforts to address them are proceeding in parallel for different file formats. BCF, for example, is the binary version of the VCF format discussed above, in which values within rows are stored in a packed binary format. Similarly, BigBed and BigWig [10] are compressed binary versions of the BED and WIG file formats, which offer efficient random access along with the ability to operate over a network.

There are significant difficulties, however, with having many different binary file formats for bioinformatics data. Each binary format requires a library and set of tools to view and process it, as it is not reasonable to expect users to decode binary files. Bindings for several different languages must also be provided, if the file format is to be widely used. Maintaining these libraries, tools and language bindings across different processor architectures and operating systems is a complex software engineering task. Maintaining this ecosystem separately for many different file formats is surely unsustainable.

Wormtable alleviates the need for these different formats and libraries, as it is flexible enough to store many different types of data. Wormtable is portable, and has been tested on big- and little-endian platforms with 32 and 64 bit word sizes, along with many operating system combinations. To take advantage of the advanced data processing features of wormtable all that is required is a conversion program, a considerably simpler task than designing and supporting a custom binary file format.

The library supports efficient access to any data stored in wormtable format, and currently provides conversion utilities for the VCF and GTF formats. The most important aspect of future development is to develop tools to convert other tabular formats such as PSL, GFF, SAM and BED to wormtable format. Such tools are not difficult to develop, since all that is required is a parser for the format in question written in Python.

Wormtable is currently limited to supporting Python, and another important aspect of future development is to create a C library along with bindings for other popular languages such as Perl, R and PHP. Wormtable does not support interval search, and so it is not straightforward to find, for example, all rows overlapping a given genomic region in GTF files. This problem has been solved several times, however, and we aim to adapt existing techniques [1,10] and incorporate them into wormtable. Compression of data can result in poor performance, but it is often necessary when volumes of data are very large. Thus, we plan on introducing optional compression of the data file in wormtable in a future release. Beyond these additions, it is difficult predict the precise direction of future development since this depends on feedback from the community. Wormtable is an open and collaborative project actively seeking feedback and contributors.

## Conclusions

The volume of data being produced in biological research is growing rapidly, but the tools available to end users to process data are still mostly based on parsing plain text. This approach is very inefficient, and leads to several undesirable outcomes. Firstly, and most obviously, a researcher’s productivity is inevitably constrained while waiting several hours for the result of a simple calculation. Without flexible indexing, working with a subset of a data file usually requires the creation of another file consisting of the subset in question, requiring extra storage and maintenance. Additionally, code quality is reduced, since testing over the entire dataset is infeasible and it is less likely that the effects of changing arbitrary analysis parameters will be systematically examined.

The classical approach to solving problems of this type is to use a relational database, which provide sophisticated data management techniques. However, relational databases are unsuitable for storing static datasets as they are complex to use and incur many unnecessary overheads. Wormtable provides the most important features of database technologies (packed binary storage of values; random access to rows; general purpose indexing) without additional complexities and overheads. Wormtable’s data model is also less rigid than relational databases, supporting, for example, columns containing a variable number of integers. Finally, wormtable is far more adaptable than a relational database. All widely used database systems are complex and adding required features (e.g. compression) would be very difficult. Adding new features to wormtable, on the other hand, is straightforward because it is far simpler and does not need to be compatible with the relational model and decades worth of existing software.

The most important aspect of wormtable is its efficiency and ease of use for end users, and we illustrated these points using some examples of VCF data. After converting a file in VCF format to wormtable using vcf2wt a user can process the data very efficiently using Python. Accessing data from rows in wormtable is many times faster than is possible by parsing rows encoded as text. This is also a very convenient way to access VCF data, since individual columns are already parsed and all that is needed is the name of the column of interest. To access regions of the genome efficiently the user simply needs to create an index on the chromosome (CHROM) and position (POS) columns using the wtadmin add command. Wormtable is not limited to VCF, but can store any form of fixed tabular data. We provide a conversion tool for the GTF format, and several others are planned or could be contributed by users.

Wormtable is not intended to replace text files as the universal interchange format for biological data. It is intended to provide a persistent data structure that can be efficiently processed and searched. Using this data structure, researchers with no knowledge of database systems can take full advantage of sophisticated data management techniques, and write straightforward code to process data efficiently. Different file formats can be handled consistently in wormtable, reducing the need for third party libraries to parse complex files and simplifying the code required to process data. Together, these advantages of increased performance and reduced code complexity can substantially increase a researcher’s productivity and ability to explore their data.

## Availability and requirements

**Project name:** Wormtable.**Project home page:** https://pypi.python.org/pypi/wormtable**Operating system(s):** POSIX.**Programming language:** C, Python.**Other requirements:** Berkeley DB 4.8 or later.**License:** GNU LGPLv3+.**Any restrictions to use by non-academics:** None.

## Competing interests

The authors declare that they have no competing interests.

## Authors’ contributions

JK conceived the project, developed wormtable source code and associated scripts and drafted the manuscript. RN wrote the tutorial and contributed to the example scripts and the manuscript. DH wrote the example scripts and vcf-utils, and contributed to vcf2wt, the tutorial and the manuscript. All authors read and approved the final manuscript.

## References

[B1] Li H **Tabix: fast retrieval of sequence features from generic TAB-delimited files** Bioinformatics 2011 27 5 718 719 10.1093/bioinformatics/btq671 21208982 PMC3042176

[B2] **IEEE standard for floating-point arithmetic** IEEE Std 754-2008 2008 1 70 doi:10.1109/IEEESTD.2008.4610935

[B3] Olson MA Bostic K Seltzer M **Berkeley, DB** Proceedings of the FREENIX Track: 1999 USENIX Annual Technical Conference 1999

[B4] Seltzer M Bostic K Brown A, Wilson G **Berkeley, DB** The Architecture of Open Source Applications, Volume 1 ISBN 978-1-257-63801-7 2012

[B5] Danecek P Auton A Abecasis G Albers CA Banks E DePristo MA Handsaker RE Lunter G Marth GT Sherry ST McVean G Durbin R 1000 Genomes Project Analysis Group **The variant call format and VCFtools** Bioinformatics 2011 27 15 2156 2158 10.1093/bioinformatics/btr330 21653522 PMC3137218

[B6] Mackay TFC Richards S Stone EA Barbadilla A Ayroles JF Zhu D Casillas S Han Y Magwire MM Cridland JM Richardson MF Anholt RRH Barrón M Bess C Blankenburg KP Carbone MA Castellano D Chaboub L Duncan L Harris Z Javaid M Jayaseelan JC Jhangiani SN Jordan KW Lara F Lawrence F Lee SL Librado P Linheiro RS Lyman RF **The**** *Drosophila melanogaster* **** genetic reference panel** Nature 2012 482 173 178 10.1038/nature10811 22318601 PMC3683990

[B7] **Drosophila Genetic Reference Panel, February 2013** [ftp://ftp.hgsc.bcm.edu/DGRP/freeze2_Feb_2013/vcf_files/freeze2.vcf.gz]

[B8] **PyVCF** [http://pyvcf.rtfd.org]

[B9] Denning PJ **The locality principle** Commun ACM 2005 48 7 19 24 10.1145/1070838.1070856

[B10] Kent WJ Zweig AS Barber G Hinrichs AS Karolchik D **BigWig and BigBed: enabling browsing of large distributed datasets** Bioinformatics 2010 26 17 2204 2207 10.1093/bioinformatics/btq351 20639541 PMC2922891

